# Hyposalivation and salivary gland histopathology in graft-versus-host disease

**DOI:** 10.3389/froh.2026.1812689

**Published:** 2026-05-08

**Authors:** Victor Tollemar, Anna Hilmer, Camilla Ivinger, Rachael V. Sugars

**Affiliations:** Department of Dental Medicine, Karolinska Institutet, Stockholm, Sweden

**Keywords:** graft-versus-host disease, histopathology, hyposalivation, immunopathology, minor salivary gland, myeloablative conditioning, oral mucosa

## Abstract

**Introduction:**

A major long-term complication following allogeneic hematopoietic cell transplantation (HCT) is graft-versus-host disease (GVHD). Oral chronic GVHD (cGVHD) is common and affects the mucosa with diagnostic lichenoid-like manifestations. Distinctive features such as hyposalivation and inflammatory histopathology of the salivary glands have been associated with both transplant regimen and GVHD. We retrospectively explored the association of hyposalivation and minor salivary gland (MSG) histopathology with the establishment and progression of GVHD.

**Methods:**

A total of 791 patient records were screened, and patients were included if salivary flow measurements were registered or if the biopsies contained both oral mucosal and MSG histopathology for comparison. In total, 63 unstimulated saliva and 123 stimulated saliva measurements were analyzed using the Kruskal–Wallis test with *post hoc* Dunn's multiple comparisons across acute, early chronic, and late chronic time phases. A total of 57 biopsies of MSG and mucosal tissue underwent histopathological grading according to NIH criteria, and 40 biopsies underwent immunohistochemical staining for CD4, CD8, CD68, and CD1a, which were analyzed using weighted kappa and Spearman's correlation.

**Results:**

Myeloablative conditioning had a significant impact on hyposalivation post-HCT (*p* < 0.0001), but no statistical difference was observed with or without acute or chronic GVHD. For all biopsies post-HCT, a moderate correlation was found between MSG and oral mucosal pathology scores (*r* 0.40), CD4 (*r* 0.69), and CD8 (*r* 0.51). Significant differences were observed across pathological phases. At cGVHD onset, a moderate to strong correlations were found with pathology score (*r* 0.73), CD4 (*r* 0.68), and CD8 infiltrate (*r* 0.84). However, at later stages, pathology score and CD8 infiltrate were non-significant.

**Discussion:**

There is limited evidence to suggest that all patients develop hyposalivation due to oral or extra-oral GVHD. MSG and oral mucosal immunopathology showed strong correlation in histopathological severity and CD8 infiltration at onset, suggesting a common initiation response. However, no correlation was found thereafter, favoring different pathophysiologies.

## 1 Introduction

Allogeneic hematopoietic cell transplantation (HCT) is widely used to treat hematopoietic cancers and non-malignant immune disorders (1). Today, HCT is often initiated with reduced-intensity conditioning (RIC) or non-myeloablative chemotherapy, whereas historically, preparative myeloablative conditioning (MAC) was administered using high-intensity chemotherapy or a combination of chemotherapy and increased doses of total-body irradiation (TBI) or fractionated TBI (2). Oral complications following HCT are numerous and often associated with lower quality of life, particularly within the first year, which include oral pain, impaired dietary performance, and swallowing difficulties (3, 4). Patients might develop oral mucositis, viral and candida infections, hyposalivation, xerostomia, and taste dysfunction. Later complications include oral disorders like caries, periodontitis, and graft-versus-host-disease (GVHD), as well as fibrosis and secondary malignancies (4). The etiology of these symptoms is multifactorial, influenced by conditioning regimen, immunosuppressive status, and disease burden.

Saliva flow rate and composition are affected by chemotherapy, with reversible and often minor damage (5). However, radiation damage is either temporary or irreversible, with fibrosis occurring within the head and neck region depending on cumulative dose and radiation field (6, 7). Different intensity protocols prior to HCT are often not fatal and are reversible, although they may manifest as symptoms and reduced function of the affected salivary glands (6, 7). Conditioning-induced inflammation in the major salivary glands has been reported to persist at least the 100 days post-HCT (8). Salivary flow is measured as unstimulated whole saliva (UWS) and stimulated whole saliva (SWS). Loss of salivary flow may be due to increasing age, particularly affecting the unstimulated capacity, but can also result from numerous disorders, diminished overall health, and medications including chemotherapy, polypharmacy, and radiation therapy (6, 9). The submandibular and parotid glands are the main contributors to whole UWS and SWS, whereas the sublingual and minor salivary glands (MSGs) contribute <10% (9). Dysfunction of the salivary glands can be objectively measured and clinically diagnosed as hyposalivation (UWS: ≤0.1 mL/min and SWS: ≤0.7 mL/min) (7). Hypofunction of MSG often leads to lack of mucosal lubrication, whereas dysfunctional stimulated capacity results in diminishing oral clearance and mastication.

GVHD is an autoimmune-like complication following HCT, caused by the donor stem cells' response to the recipient's tissues, and symptoms range from mild to severe. Beside classic acute GVHD (aGVHD) that onsets within 100 days, aGVHD also includes persistent and recurrent late aGVHD (>100 days), as well as de novo aGVHD (initiated >100 days) (10). Although aGVHD seldom occurs in the mouth, the oral cavity is one of the most commonly affected organs in cGVHD, with historical prevalence ranging from 45% to 83% (4, 11). According to the National Institute of Health (NIH) diagnostic criteria, oral mucosal lichenoid-like manifestations are considered clinically diagnostic for oral cGVHD (12). There are no diagnostic criteria to establish oral salivary gland cGVHD, as xerostomia, mucoceles, mucosal atrophy, pseudomembrane, and ulcers are only distinctive features and insufficient to establish a diagnosis alone (12). Criteria for histopathological definitions have been proposed by the NIH and diagnostic grades have been proposed to establish verification of oral mucosal and salivary gland pathology (13–15). To facilitate relevant decision-making in diagnosing salivary gland cGVHD, a cutoff for clinical UWS (≤0.2 mL/min) was suggested, which was found to be associated with lacrimal cGVHD rather than oral mucosal cGVHD (16, 17).

Within our saliva cohort, we aimed to map salivary flow rates and hyposalivation in both the short- and long-term post-HCT and to associate prevalence with GVHD subtypes. Furthermore, we investigated our biopsy cohort for immunopathological correlation and agreement between oral mucosal and MSG pathophysiology, to verify the lack of clinical overlap. We hypothesized that salivary flow rates would be affected by the MAC intensity during early time phases post-HCT, whereas hyposalivation in the chronic period post-HCT would be dependent on different GVHD subtypes and associated pathophysiologies in salivary gland or mucosal tissue structures.

## 2 Materials and methods

### 2.1 Ethical considerations

This study was approved by the Swedish Ethical Review Authority (DNR 2019-01259 and DNR 2013/1241-31/1) and conducted in accordance with the ethical principles of the Declaration of Helsinki.

### 2.2 Retrospective study cohort and parameters

**Figure 1:**
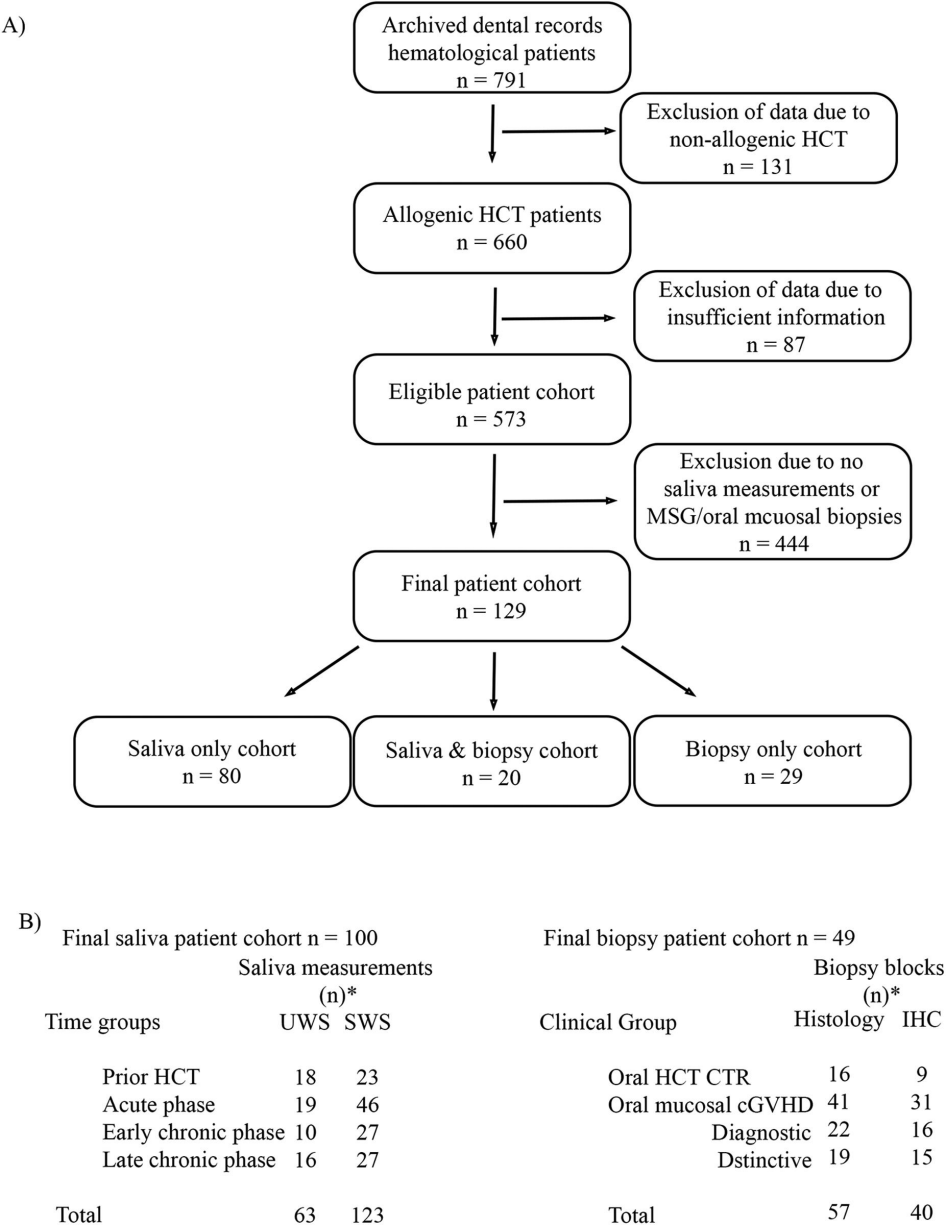
**(A)** Archived dental records of 791 hematological patients were screened for allogenic cell transplantation, detailed patient information, GVHD diagnosis, and whether saliva and/or biopsy samples had been retrieved. An eligible cohort of 129 patients was identified, with some patients providing saliva measurements only, oral mucosa biopsies including minor salivary glands (MSG), or both. **(B)** One hundred patients provided information on saliva, grouped according to the time phase of diagnosis, resulting in a total of 219 saliva samples. Patients provided unstimulated (UWS) and/or stimulated (SWS) saliva samples. Forty-nine patients provided a total of 57 biopsies that were classified according to whether they were considered oral HCT controls or oral cGVHD with a clinical diagnostic or distinctive classification. Biopsies were histologically stained and graded (G0-GIV) according to the classifications of Tollemar et al. (14) and Tollemar et al. (15) for oral mucosal and MSG cGVHD, respectively. Immunohistochemistry (IHC) for CD4, CD8, CD68, and CD1a was also performed on 40 biopsy samples when tissue was available. *Many patients provided multiple samples across different time points and clinical stages through their diseases course, and mean values were calculated for each patient in such cases.

This retrospective cohort from the Oral and Maxillofacial Surgery Clinic at Karolinska University Hospital, archived at the Department of Dental Medicine, Karolinska Institutet, has been previously described (14, 15, 18). A total of 791 clinical files archived from individuals selected for HCT between 1977 and 2011, who had been referred for dental examination, were screened by two independent reviewers to identify cases with saliva measurements and MSG–oral mucosal biopsy analysis (Figure 1A). UWS and SWS measurements were routinely collected over 5 min. Biopsies were commonly collected every 3 months in the first year post-HCT, and on an individual basis thereafter. Oral cGVHD was defined based on NIH diagnostic criteria, with distinctive manifestations validated by pathological grade of “possible or likely” cGVHD (12, 13).

#### 2.2.1 Saliva cohort

Patient data were included if one or more measurements of salivary flow had been recorded prior to or following HCT (Figure 1B). For patients with multiple measurements within each time frame, values were adjusted to a mean. Therefore, the final dataset consisted of 63 UWS and 123 SWS measurements from 100 patients (Figure 1B). Saliva measurements prior to HCT constituted the control group, whereas patients who received RIC were used as controls post-HCT. Saliva measurements from patients treated with MAC were further analyzed based on overall GVHD diagnosis (none or a/cGVHD). The saliva cohort was divided into three consecutive clinical groups based on time and GVHD post-HCT: The acute time phase enrolled patients without aGVHD during the first 3 months, as well as aGVHD patients prior to their cGVHD diagnosis. The chronic time phase was divided into early and late to discriminate our data against previous research results before and after the first year of HCT. Early phase included measurements obtained 3 months post-HCT, or post-cGVHD diagnosis, but within the first year after transplant. Late phase included measurements obtained after the first year of HCT. Of note, one patient within the acute time phase was defined as non-aGVHD since this patient developed late-stage aGVHD at day 144, 90 days after the saliva collection. Furthermore, one patient in the chronic phase was considered non-cGVHD, as this patient developed cGVHD 8 years post-HCT.

#### 2.2.2 Biopsy cohort

Biopsies were retrieved from Stockholm′ s Medicine Biobank (SMB) to assess the MSG and oral mucosal cGVHD immunopathological profiles (Figure 1B) (14, 15, 18). Forty-nine patients were included for intraindividual immunopathology correlation, as both oral mucosa and MSG tissue were available and had been pathologically scored and graded according to the NIH formalized protocol (*n* = 57 biopsies). In addition, the biopsies had been immunohistochemically profiled for CD4, CD8, CD68, and CD1a (*n* = 40 biopsies) (14, 15, 18). Histopathological grading data consisted of pathology scores for MSG (0–16) and oral mucosa (0–19), as well as histopathological grades (G)0-IV. Clinical subgroups were defined based on conditioning and oral mucosal status. Oral HCT controls (non-cGVHD) had healthy mucosal structures, whereas oral mucosal cGVHD was analyzed as a consolidated group or based on clinical distinctive (non-verified lichenoid cGVHD) or diagnostic (lichenoid-like cGVHD) manifestations. For intrabiopsy comparisons, multiple data outputs from the same patient within specified clinical groups were adjusted to a single mean value.

### 2.3 Statistics

Statistical analyses were performed using Prism 10 (GraphPad Software, La Jolla, CA, USA). The Kruskal–Wallis test with *post hoc* Dunn's multiple comparisons was applied to the clinical groups of saliva measurements prior to and following HCT. Descriptive data and statistics were reported as range, mean rank, standard deviation (SD), and standard error (SE). Significance was defined as *p*-value ≤ 0.05. Cohens weighted Kappa (κ) was performed of intrabiopsy accuracy between the NIH formalized grade no- (G0-I), possible- (GII) or likely- (GIII-GIV) GVHD, between MSG and oral mucosa tissue (14, 15). Correlation of intrabiopsy pathology scores (MSG: 0–16; oral mucosa: 0–19) was calculated using Spearman's correlation, with *p*-value ≤ 0.05 considered significant. Immunohistochemical mean pixel area for CD4, CD8, CD1a, and CD68 stained antibodies has been described previously (15, 18). The presence of CD4, CD8, CD1a, and CD68 immunolocalization in MSG and oral mucosa was correlated using Spearman's statistics to evaluate associations between the two affected tissue sites by cGVHD. 95% confidence intervals (CIs) were reported, and *p*-value ≤ 0.05 was considered significant.

## 3 Results

### 3.1 Patient characteristics

In the full HCT cohort, 129 patients were included. Clinical registry characteristics from Karolinska University Hospital are presented in Table 1 and Figures 1A,B. Twenty of these patients overlapped and were included in both the saliva and biopsy cohorts. For the salivary flow analysis, one prior-HCT UWS patient was excluded due to uncertainty regarding saliva collection. Thus, the full cohort consisted of 99 patients, with 62 UWS and 123 SWS measurements. Distribution of saliva measurements across time groups and intervals is shown in Figure 1B and Supplementary SI 1. Oral cGVHD was diagnosed in 41 patients; 28 of these had saliva measurements and 30 had biopsies of MSG and oral mucosal tissue for intrapathology comparison. To our knowledge, two patients with oral cGVHD later developed oral squamous cell carcinoma.

**Table 1:** Clinical characteristics of HCT patients.

Characteristics Cohort	*N* (% or range)		
	Whole	Saliva	Biopsy
No. of patients	129	100	49
Unstimulated whole saliva (UWS)	63	63	-
Stimulated whole saliva (SWS)	123	123	-
Histopathological assessed biopsies	57	-	57
Immunohistochemical assessed biopsies	40	-	40
Age and gender			
Median age (years) at HCT	31 (5–59)	29.5 (5–59)	26 (5–53)
Adults (≥18 years)	35 (18–59)	35 (18–59)	32 (18–53)
Children (<18 years)	13.5 (5–17)	14 (5–17)	13 (5–17)
Ratio male/female	77/52 (60/40)	57/43 (57/43)	36/13 (73/27)
Year of HCT			
1979–1989	67 (52)	51 (51)	35 (71)
1990–1998	57 (44)	44 (44)	14 (29)
2008	3 (2)	3 (3)	0
Unknown	2 (2)	2 (2)	0
Donor			
HLA identical related	95 (74)	72 (72)	41 (84)
Matched unrelated/related	23 (18)	20 (20)	4 (8)
Mismatched (related, allele unrelated)	10 (8)	7 (7)	4 (8)
Unknown	1 (0)	1 (1)	0
Gender (unmatched/matched/unknown)	73/55/1 (57/43/0)	54/45/1 (54/45/1)	29/20/0 (59/41/0)
Disease			
Acute leukemia	61 (47)	52 (52)	20 (41)
Chronic leukemia	39 (30)	29 (29)	12 (25)
Aplastic anemia	13 (10)	9 (9)	9 (18)
Multiple myeloma	5 (4)	1 (1)	4 (8)
Lymphoma	5 (4)	4 (4)	1 (2)
Other (MDS/MPS, MF, Metab)/unknown	5/1 (4/1)	5/0 (5/0)	2/1 (4/2)
Conditioning regimen			
Myeloablative	114 (88)	89 89)	40
TBI10 + Cy ± Etoposide	97 (85)	82 (92)	30 (75)
TBI7.5 + Cy	5 (4)	2 (2)	3 (8)
Fractionated TBI+Cy ± Melphalan	2 (2)	0	2 (5)
Busulfan + Cy	9 (8)	4 (5)	5 (12)
Unspecified TBI	1 (1)	1 (1)	0
Reduced intensity	14 (11)	10 (10)	9 (18)
Cy	12 (86)	8 (80)	9 (100)
Fractionated TBI+Cy + Fludarabine	2 (14)	2 (20)	0
Unknown	1 (1)	1 (1)	0
GVHD prophylaxis			
CsA+MTX/MTX/CsA	65/27/25 (51/21/19)	47/25/21 (47/25/21)	20/11/13 (41/22/27)
Other (Tac + Sir, TcD, CsA + Pred)/unknown	8/4 (6/3)	5/2 (5/2)	3/2 (6/4)
HCT source			
BM/PBSC/unknown	117/10/2 (91/8/1)	90/8/2 (90/8/2)	46/3/0 (94/6/0)
aGVHD grade			
0	33 (26)	22 (22)	15 (31)
1	67 (52)	54 (54)	26 (53)
2	18 (14)	13 (13)	8 (16)
3	7 (5)	7 (7)	0
4	3 (2)	3 (3)	0
Unknown	1 (1)	1 (1)	0
cGVHD score			
No	51 (40)	41 (41)	12 (25)
Mild	59 (46)	46 (46)	24 (49)
Moderate	12 (9)	6 (6)	11 (22)
Severe	5 (4)	5 (5)	2 (4)
Unknown	2 (1)	2 (2)	0
Oral cGVHD			
Yes	41 (32)	28 (28)	30 (61)
No	88 (68)	72 (72)	19 (39)

HCT, hematopoietic cell transplantation; MDS, myelodysplastic syndrome; MPS, myeloproliferative syndrome; MF, Myelofibrosis; Metab, metabolic disorders; TBI, Total body irradiation; Cy, Cytoxan; CsA, cyclosporine A; MTX, methotrexate; Tac, tacrolimus; Sir, sirolimus; TcD, T-cell depletion; BM, bone marrow; PBSC, peripheral blood stem cell.

### 3.2 Hyposalivation attributed to conditioning intensity

**Figure 2:**
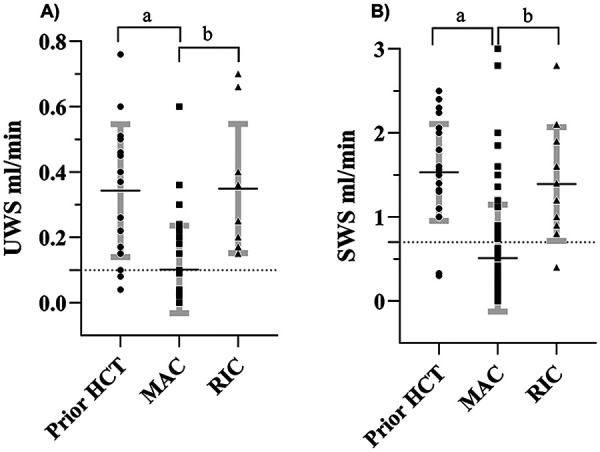
Whole-cohort analysis of UWS (*n* = 62) **(A)** and SWS (*n* = 123) **(B)** salivary flow measurements. Hyposalivation in MAC-treated patients (square) was significantly diminished compared to prior-HCT (circle) and RIC controls (triangle). Data are displayed as mean rank flow rate mL/min with standard deviations (SD) provided. Standard error is outlined in bold gray error bars. Dashed line indicates hyposalivation cutoff (UWS: ≤0.1 mL/min and SWS: ≤0.7 mL/min). Statistical significance is indicated by a: *p* < 0.0001 and b: *p* < 0.005. UWS, unstimulated whole saliva; SWS, stimulated whole saliva.

Twenty-three patients had saliva measurements prior to allogenic HCT. Seventeen prior-HCT measurements of UWS were displayed with a mean flow rate of 0.34 mL/min, ±0.20 SD, whereas 23 prior-HCT SWS flow rates were found with a mean of 1.53 mL/min, ±0.58 SD (Figure 2). Hyposalivation was observed in 18% of the UWS flow rates (≤0.1 mL/min) and 9% of SWS (≤0.7 mL/min) (7). Eighty patients were further analyzed post-HCT, of whom 72 had received MAC, with 35 UWS and 89 SWS measurements analyzed. Overall, saliva measurements from patients with MAC showed significantly (*p* < 0.0001) lower mean UWS (0.10 mL/min, ±0.13 SD) and SWS (0.51 mL/min, ±0.58 SD) compared with prior-HCT values (Figure 2). Eight patients had RIC-induced flow rates, with 10 UWS and 11 SWS measurements. RIC-induced UWS (0.35 mL/min, ±0.20 SD) and SWS (1.39 mL/min, ±0.68 SD) flow rate did not significantly differ from prior-HCT values, but were significantly higher than MAC (UWS, *p* = 0.006 and SWS, *p* = 0.0004, respectively) (Figure 2). Eighteen patients were children, with a total of 36 saliva measurements (15 UWS and 21 SWS). Non-significant differences were seen between the different groups with child salivary flow rates. However, MAC-induced mean UWS (0.08 mL/min, ±0.11 SD) and SWS (0.27 mL/min, ±0.25 SD) showed diminishing volumes than the MAC adult population and were significantly lower (*p* < 0.0001) compared with prior-HCT adults (Supplementary SI 2). Hence, further analysis continued as a mixed whole cohort.

### 3.3 Myeloablative-induced hyposalivation over time and presence of GVHD

**Figure 3:**
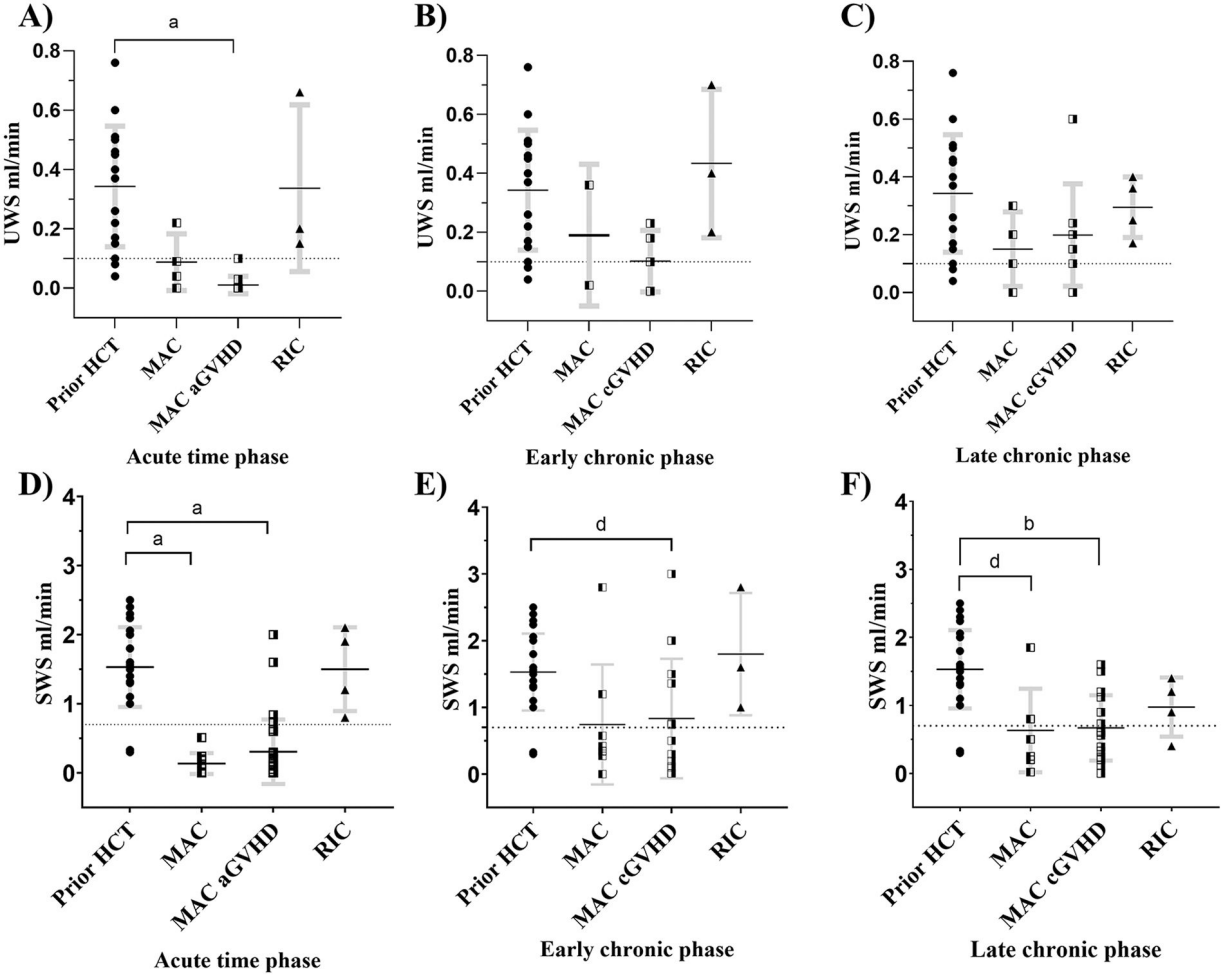
Changes in UWS and SWS salivary flow rates across acute, early chronic, and late chronic phases. The only significance found in MAC UWS subgroups was seen in the acute time phase **(A)** In SWS rates, both MAC patients with and without a/cGVHD showed significantly lower flow rates than prior-HCT, except for MAC patients without cGVHD in the early chronic phase **(D–F)**. Data are displayed as mean rank flow rate mL/min with standard deviations. Standard error is outlined in bold gray error bars. Dashed line indicates hyposalivation cutoff (UWS: ≤0.1 mL/min and SWS: ≤0.7 mL/min). Statistical significance is indicated by a: *p* < 0.0001, b: *p* < 0.005 and d: *p* < 0.05. UWS, unstimulated whole saliva; SWS, stimulated whole saliva. Statistical comparisons in patients with RIC measurements were not plotted in the acute, early chronic, and late chronic time phases due to lack of reliability and are only visually expressed as groups.

Changes to MAC-induced salivary flow rates in the acute phase displayed significantly (*p* < 0.0001) lower flow rates in 16 UWS measurements (0.03 mL/min) and 42 SWS flow rates (0.26 mL/min), compared to prior HCT (Figures 3A,D, Supplementary SI 1). These findings were independent of aGVHD status (UWS: 0.01 mL/min, and SWS: 0.31 mL/min), as an overall diagnosis did not significantly influence flow rates compared to patients without aGVHD (UWS: 0.1 mL/min, and SWS: 0.13 mL/min) (Figures 3A,D, Supplementary SI 1). Overall, 94% of UWS (≤0.1 mL/min) and 90% of SWS (≤0.7 mL/min) met criteria for clinical hyposalivation in the acute time phase (7).

In the early chronic phase, MAC patients continued to show lower flow rates compared with prior HCT. Twenty-four SWS flow rates displayed a significantly lower mean of 0.81 mL/min compared to prior-HCT, but was significantly increased compared to the MAC acute phase (Figure 3E, Supplementary SI 1). Overall, 50% of SWS flow rates met hyposalivation criteria. Only seven measurements of UWS were registered in the early chronic phase, with a non-significant mean value of 0.13 mL/min, though 57% were classified as hyposalivation (Figure 3B). No statistical differences in UWS or SWS were found between patients with or without cGVHD.

In the late chronic phase, 12 MAC UWS measurements showed hyposalivation in 33% of cases, but flow rates were again non-significant (0.18 mL/min) compared with prior HCT. A 61% hyposalivation with decreased SWS flow rates (0.66 mL/min) was observed in 23 measurements in relation to prior HCT. Overall, cGVHD patients (UWS: 0.20 mL/min and SWS: 0.67 mL/min) did not differ significantly from non-cGVHD patients (UWS: 0.15 mL/min and SWS: 0.63 mL/min) in MAC recipients in late chronic phase (Figures 3C,F, Supplementary SI 1). No UWS flow rates in RIC patients displayed hyposalivation, regardless of time phases post-HCT (Figures 3A–C, Supplementary SI 1). Similar trends were observed for SWS flow rates, despite one hyposalivation measurement being registered in the late chronic phase (Figure 3F, Supplementary SI 1). Statistical comparisons between study groups post-HCT are detailed in Supplementary SI 1.

### 3.4 Hyposalivation over time and presence of GVHD

**Figure 4:**
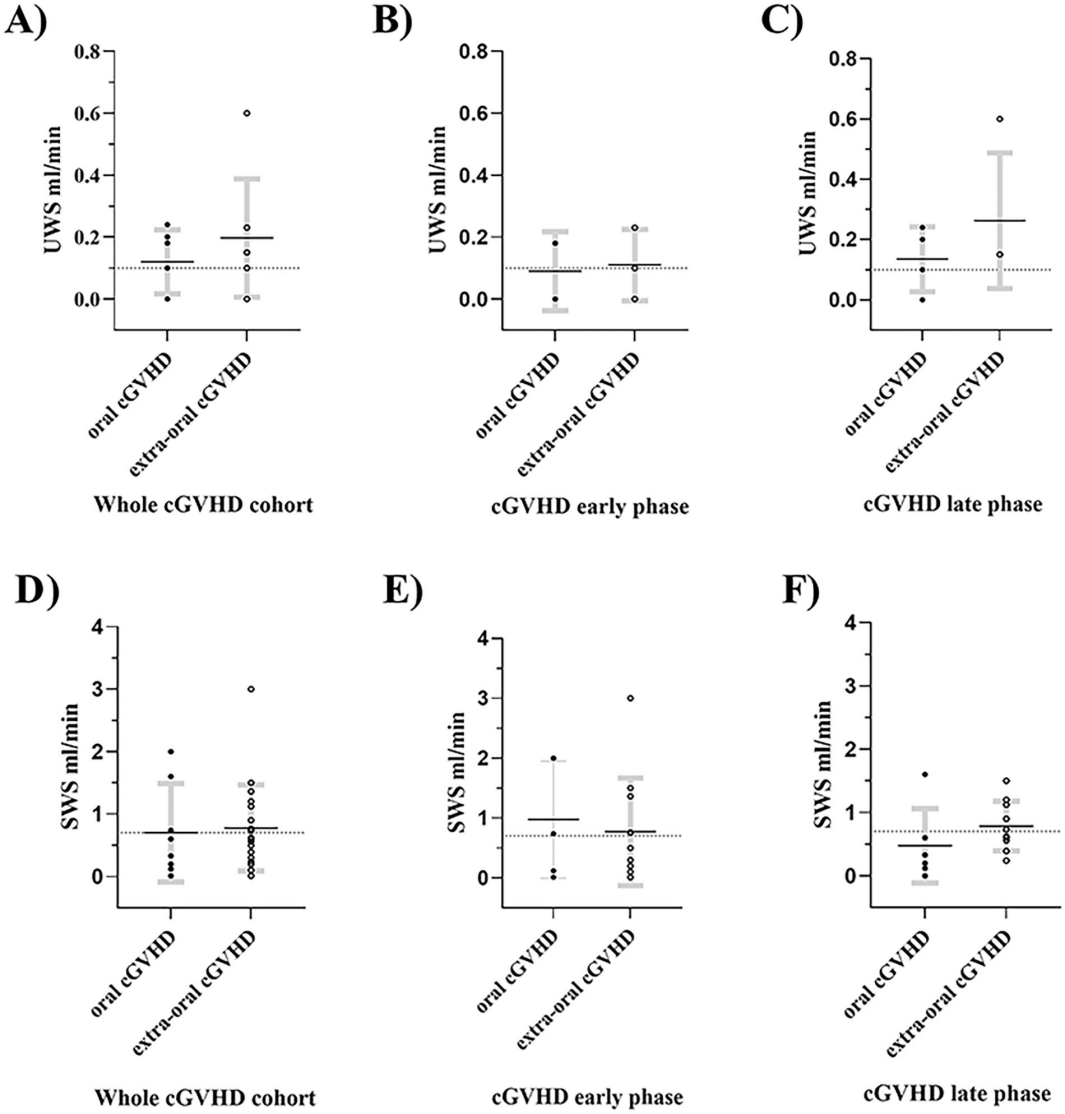
Changes in UWS and SWS in oral cGVHD and extraoral cGVHD with respect to the whole cGVHD cohort **(A and D)**, early chronic cGVHD **(B and E)**, and later chronic cGVHD **(C and D)**. No statistical significance was found between the groups. Data are displayed as mean rank flow rate mL/min with standard deviations. Standard error is outlined in bold gray error bars. Dashed line indicates hyposalivation cutoff (UWS: ≤0.1 mL/min and SWS: ≤0.7 mL/min). UWS, unstimulated whole saliva: SWS, stimulated whole saliva.

Twenty patients had more than one SWS measurement registered over different time periods, whereas only seven UWS patients had similar data. These patients were analyzed using paired analysis between across time periods of prior-HCT, acute, chronic early, and chronic late phases (Supplementary SI 3 and 4). The paired data of SWS reflected the distribution of the whole cohort, with visible partial recovery in the early and late chronic phases; however, a substantial recovery to baseline levels prior to HCT was not observed (Figures 3D–F, Supplementary SI 3). The UWS data were more dynamic, with patient-dependent recovery (SI 4). The association with oral cGVHD was studied in a subcohort of saliva measurements from patients diagnosed with oral mucosal cGVHD (*n* = 11) compared with those who had extraoral cGVHD (*n* = 18). Both cGVHD groups had mild global cGVHD (>80%), but one oral cGVHD patient had moderate disease and one developed severe disease. In the extraoral cGVHD cohort, three patients were diagnosed with moderate cGVHD. In this subanalysis, a total of 13 UWS measurements (*n* = 6 oral cGVHD 0.12 mL/min and *n* = 7 extraoral cGVHD 0.2 mL/min) and 32 SWS measurements (11 oral cGVHD 0.70 mL/min and 21 extraoral cGVHD 0.78 mL/min) were identified post-HCT but showed non-significant differences (Figure 4).

### 3.5 Immunopathological association post-allogeneic transplantation

The association between MSG and oral mucosal cGVHD immunopathology showed moderate correlations in pathology scores (*n* = 57: *r* 0.40, *p* < 0.005), CD4 T-helper cell localization (*n* = 38: *r* 0.69, *p* < 0.0001), and CD8 T-suppressor cell localization (*n* = 37: *r* 0.51, *p* < 0.005) in all samples post-HCT (Table 2). However, the accuracy of pathological GVHD diagnostics of “no” (G0-GI), “possible” (GII) and “likely” (GIII-GIV) showed only fair agreement (k 0.29), and lack of correlation in CD68 monocytic immunolocalization (*n* = 40: *r* −0.14) and CD1a dendritic immunolocalization (*n* = 38: *r* −0.09). Differences were observed depending on the type of conditioning. Post-HCT MAC patients did not deviate from the trends observed for all post-HCT samples, whereas RIC data showed an increased association of pathology scores (*n* = 14: *r* 0.69, *p* < 0.01) and CD4-cell infiltrate (*n* = 9: *r* 0.60, ns), but CD8 data did not correlate (*n* = 9: *r* 0.17, ns) (Table 2).

**Table 2:** Alignment of minor salivary glands (MSG) and oral mucosal immunopathology post-HCT, with respect to conditioning and cGVHD manifestations.

Group	Pathology (*r*)	Pathology (*k*)	CD4 (*r*)	CD8 (*r*)	CD68 (*r*)	CD1a (*r*)
Post-HCT	**0.40** (0.15–0.60) **b. 0.0019**	0.29 (Fair)	**0.69** (0.46–0.83) **a. <0.0001**	**0.51** (0.21–0.72) **b. 0.0014**	−0.14 (−0.44–0.19) p. 0.3880	−0.09 (−0.40–0.25) p. 0.5996
RIC	**0.69** (0.24–0.90) **c. 0.0075**	0.50 (Moderate)	0.60 p. 0.1323	0.17 p. 0.6777	−0.12 p. 0.7756	−0.73 p. 0.0714
MAC	0.30 (−0.01–0.56) p. 0.0524	0.23 (Fair)	**0.70** (0.45–0.85) **a. <0.0001**	**0.58** (0.25–0.79) **b.** **0.0012**	−0.20 (−0.53–0.18) p. 0.2811	0.07 (−0.31–0.43) p. 0.7023
Oral HCT control	0.40 (−0.13–0.76) p. 0.1223	0.13 (Slight)	**0.82** **d.** **0.0108**	0.43* p. 0.3536	−0.14 p. 0.7165	−0.16* p. 0.7500
Oral mucosal cGVHD	**0.37** (0.06–0.62) **d.** **0.0164**	0.32 (Fair)	0.68* (0.41–0.84) **a. <0.0001**	0.56* (0.25–0.77) **b.** **0.0011**	−0.12 (−0.46–0.25) p. 0.5174	−0.05* (−0.41–0.33) p. 0.7942
Diagnostic	0.36 (−0.09–0.68) p. 0.1035	0.38 (Fair)	0.58* (0.08–0.85) **d. 0.0252**	0.41* (−0.14–0.77) p. 0.1262	−0.16 (−0.62–0.38) p. 0.5502	−0.15 (−0.61–0.39) p. 0.5783
Distinctive	0.42 (−0.05–0.74) p. 0.0720	0.33 (Fair)	0.71* (0.28–0.91) **c. 0.0054**	**0.69** (0.26–0.89) **c. 0.0057**	−0.15 (−0.63–0.40) p. 0.5828	0.05* (−0.51–0.58) p. 0.8634

HCT, allogeneic hematopoietic cell transplantation; RIC, reduced intensity conditioning; MAC, myeloablative conditioning.

Intrabiopsy statistics are displayed as Spearman's correlation coefficient (*r*) between MSG (score 0–16) and oral mucosal (score 0–19) (14, 15). Agreement of pathological diagnostics of “no” (G0-GI), “possible” (GII), and “defined” (GIII-GIV) with weighted kappa (*k*) (14, 15). A correlation coefficient (*r*) represents alignment of mean pixel area of immunohistochemically localized CD4 T-helper cells, CD8 T-cytotoxic cells, CD68 monocytic cells, and CD1a dendritic cells within MSG and oral mucosa (15, 18). Statistically significant data (*r*) are in bold with 95% confidence interval (CI), computed by an approximation for groups with *n* > 10, as well as exact *p*-value and associated letters a: *p* < 0.0001; b: *p* < 0.005; c: <0.01; and d: *p* < 0.05. Kappa agreements are displayed by *k*-numbers as well as cutoff interpretation: 0.01–0.20: Slight; 0.21–0.40: Fair; 0.4–0.60: Moderate; 0.61–0.80: Substantial; and 0.81–1.00: Perfect.

* Stained slides for immunohistochemistry (IHC) were excluded due to poor quality for oral mucosal cGVHD: (*n* = 2) CD4, (*n* = 1) CD8 and (*n* = 1) CD1a. Oral HCT controls: (*n* = 2) CD8 and (*n* = 1) CD1a. Distinctive oral mucosal cGVHD: (*n* = 1) CD4 and (*n* = 1) CD1a. Diagnostic oral mucosal cGVHD: (*n* = 1) CD4 and (*n* = 1) CD8. Oral mucosal cGVHD 0–3 months: (*n* = 1) CD8 and (*n* = 1) CD1a. Oral mucosal cGVHD >3 months: (*n* = 2) CD4.

### 3.6 Salivary gland immunopathology association to oral cGVHD

To understand the associations of immunopathological features in the context of oral cGVHD, we investigated correlations and agreement between MSG and oral mucosal biopsies in detailed clinical subgroups (Table 2, Supplementary SI 5). Intraindividual association between MSG and oral mucosa did not differ considerably when comparing the whole post-HCT cohort against oral HCT controls or oral mucosal cGVHD analyzed as a consolidated group (Table 2, Supplementary SI 5). The distinctive oral mucosal cGVHD subgroup was found with the highest association in pathology score (*n* = 19: *r* 0.42, ns), accuracy of pathological GVHD diagnostics (*n* = 19: *k* 0.33), and alignment of CD4 (*n* = 15: *r* 0.71, *p* < 0.01) and CD8 (*n* = 15: *r* 0.69, *p* < 0.01). Among these, CD4 immunolocalization showed the strongest association, with significant moderate-to-strong correlations across all clinical groups (*r* > 0.58, *p* < 0.05) (Table 2, Supplementary SI 5).

**Figure 5:**
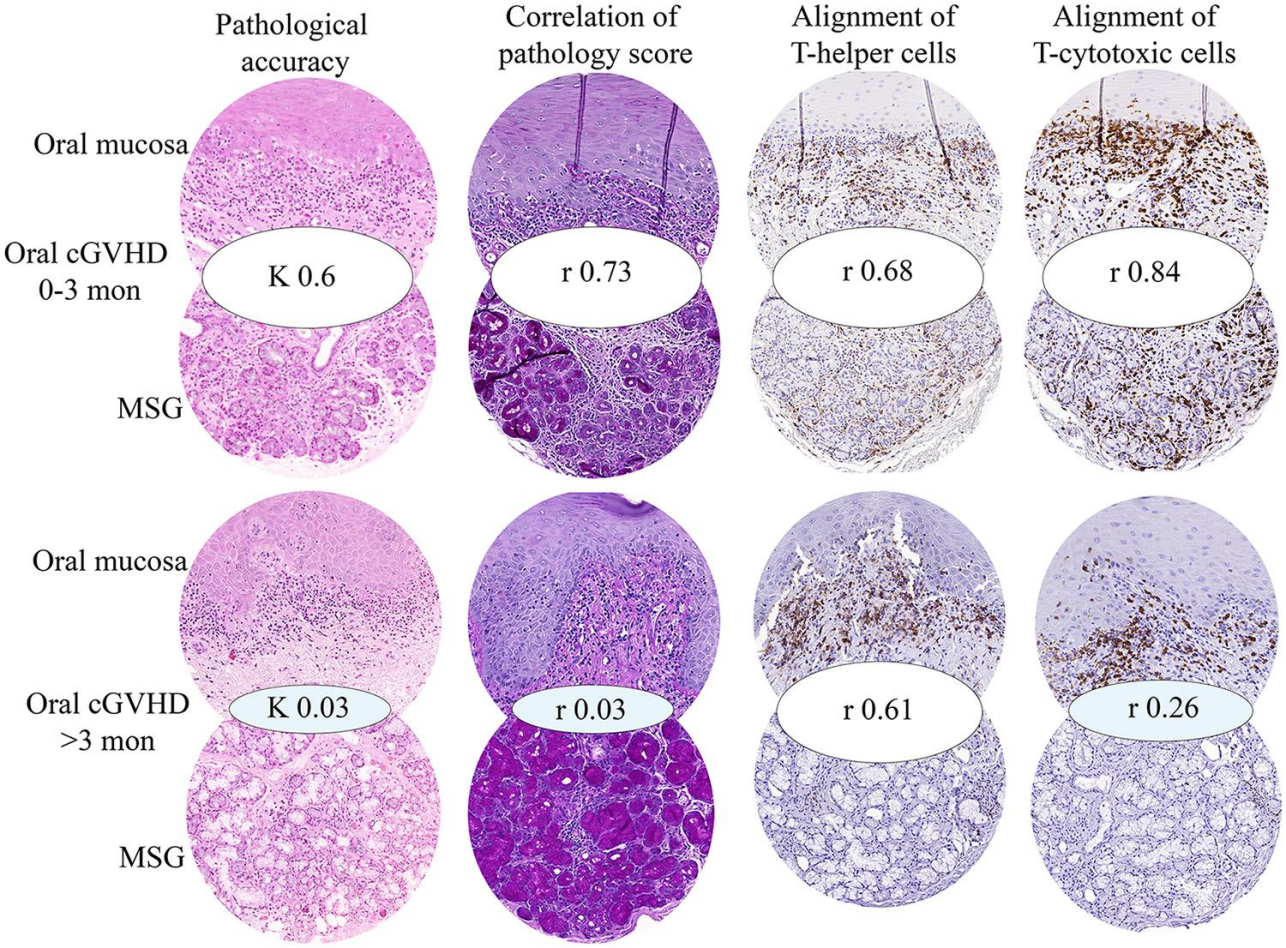
Immunopathological association between oral mucosa and MSG pathophysiology at cGVHD onset [0–3 months (mon)] and cGVHD progression (>3 mon). Pathological accuracy showed moderate–substantial agreement at onset (*k* 0.6) but negligible during progression (*k* 0.03), analyzed by weighted kappa agreement between G0-GI, GII, and GIII-GIV. Pathological score (oral mucosa 0-19 and MSG 0-16) was strongly correlated at onset (*r* 0.73) but absent at cGVHD progression *(r* 0.03). Alignment of mean pixel area for CD4 and CD8 immunolocalization was strongly associated between oral mucosa and MSG during cGVHD onset (*r* 0.68 and *r* 0.84, respectively). As cGVHD progressed, CD8 correlation was diminished (*r* 0.26) but CD4 remained present (*r* 0.61). Circled histological and immunohistochemical images represent pathological accuracy (hematoxylin and eosin), correlation of pathological score (periodic acid Schiff), alignment of T-helper cells (CD4), and alignment of T-cytotoxic cells (CD8).

The association between MSG and oral mucosa immunopathology was observed with large differences within the pathobiological phases of oral cGVHD and disease duration (Figure 5). At cGVHD onset (0–3 months), strong and significant correlations were found with pathology score (*r* 0.73, CI 0.41–0.89, *p* = 0.0003) and CD8 infiltrate (*r* 0.84, CI 0.55–0.95, *p* = 0.0003). For the first time, CD68 and CD1a displayed positive but weak non-significant associations, whereas CD4 remained moderately correlated (*r* 0.68, CI 0.24–0.89, *p* = 0,0068) (Figure 5). As cGVHD progressed (>3 months), CD4 infiltrate remained moderately correlated (*r* 0.61, CI 0.13–0.86, *p* = 0.0178), with 39% of data points confirming GVHD (GIII-IV) in MSG and oral mucosa (SI 5). However, only 14% of these confirmations overlapped, with the remainder showing absent or weak agreement in pathology scores, pathological diagnostic grades, and CD8 infiltrate (Figure 5, Supplementary SI 5).

## 4 Discussion

This retrospective cohort study was examined short- and long-term changes in salivary flow rate in allogenic HCT recipients, with emphasis on the association with GVHD. Historically, salivary hypofunction has been widely studied and linked to conditioning intensity during the early months following HCT (19). Initial research found GVHD damage in the salivary glands and associated clinical hyposalivation to patients with cGVHD (20, 21). However, although sicca symptoms are common in patients with GVHD, more recent research has distinguished between oral mucosal cGVHD and salivary gland cGVHD based on their low clinical overlap (16, 17). Hence, our study sought to validate the clinical differences in GVHD pathophysiologies and investigate the immunopathological subclinical association between oral and salivary gland GVHD.

Prior to HCT conditioning, patients were presenting with clinical hyposalivation in our cohort. SWS hyposalivation has traditionally been reported with increasing incidence (up to 56% prior HCT) (22–27). UWS levels have been less extensively studied in large cohorts, but reports suggest a range from none to 34% hyposalivation in prior-HCT patients (3, 8, 24, 26, 27). Different hematological conditions and treatment protocols may affect salivary gland function differently both prior to and following HCT, warranting further investigation (22, 26). Our MAC cohort consistently presented with >90% hyposalivation in the acute phase, with gradual improvement into the early and late chronic phases. This is comparable with the overall trend of persistent SWS hyposalivation up to 24 months post-HCT, alongside gradual improvement directly following HCT over time (22, 23, 28). UWS flow rates are also less well studied post-HCT, but our data of acute and early chronic phases are consistent with prior reports suggesting recovery, though prevalence of hyposalivation seems to be more patient-depended (3, 26). UWS data in the late chronic phase suggest a continuing hyposalivation compared to prior-HCT, which contrasts with findings of a recent 5-year follow-up study (26, 29). However, long-term cohorts have small sample sizes and lack statistical significance. Interestingly, patients with lower volumes and persistent hyposalivation have been documented >11 years post-HCT compared with their sibling donors (30).

Diminishing salivary function without substantial recovery is often observed following radiation treatment, particularly when irradiation involves the head and neck region (7, 31). However, treatment involving ≤25 Gy has been proposed as a threshold for a complete clinical recovery of salivary function (7, 31). TBI-associated HCT is therefore associated with regained salivary function, in line with our findings and those of others (26, 28, 29). Furthermore, irradiation has been recently reported as a non-significant risk factor for conditioning-induced hyposalivation, suggesting that distinguishing between MAC and non-myeloablative conditioning may be more appropriate than simply considering if TBI was administered or not to understand hyposalivation risk (26, 28). However, the present investigation is in line with other studies suggesting that MAC regimens affect salivary dysfunction. This is apparent with UWS during the acute time phase and with SWS during both the acute and chronic phases (22, 26, 28). Of note, multivariable analyses have also reported non-significant differences in SWS hyposalivation when comparing conditioning intensity, hematological disorder, and other transplant-related factors, such as graft type and medications (23, 28). Increased hematological disease burden, mortality, cGVHD, number and types of medication, and female gender have been suggested as contributors to hyposalivation post-HCT, but findings remain inconclusive across different research cohorts (22–26).

Salivary dysfunction may lead to subjective complaints and increased oral pathologies, such as candida infections, caries, and periodontitis (4). A recent systematic review rejected the scientific evidence for caries and periodontitis development in HCT survivors, which has been confirmed by a prospective 2-year follow-up (3, 32). However, case series reports post-HCT have described extensive caries progression, most probably associated with morbid oral cGVHD in both the MSG and mucosa (4, 33, 34). SWS hyposalivation at 3 and 12 months post-HCT has been proposed as a possible risk marker for increased caries and number of dental treatments (27, 29). Interestingly, neither UWS nor pH of UWS/SWS has been significantly associated with caries complications post-HCT, emphasizing the importance of future research to explore GVHD as a risk predictor to guide caries prophylaxis and management (27). Large-scale prospective studies on UWS are still needed. This is important as UWS appears to be strongly linked to subjective complaints of xerostomia and quality of life, whereas SWS is important for oral functions, such as mastication, swallowing, and caries prevention (22, 25, 27, 29).

Findings of hyposalivation, altered salivary protein content, and reduced antioxidant protection have been reported in patients with cGVHD, particularly those with severe disease profiles (21, 25, 35). In the present study, most of our GVHD patients presented with an overall mild disease profile, with no apparent association with hyposalivation. However, in agreement with other reports, our study found non-significant differences in salivary flow rates and hyposalivation between patients with overall/oral cGVHD and those without (17, 25, 26, 35, 36).

Early histopathological studies by Sale et al. reported substantial to perfect agreement between MSG and oral mucosal verification of overall cGVHD activity (37). Post-HCT, we observed a significant but weaker association for pathology score, CD4 T-helper cell, and CD8 T-suppressor cell infiltrate. Subsequent studies reported that MSG biopsies showed higher accuracy in confirming overall cGVHD compared with oral mucosal biopsies (38–40). We and others have also reported that subclinical cGVHD activity might be present in both MSG and oral mucosa, particularly during cGVHD onset (18, 41, 42). This aligns with our findings at cGVHD onset, where MSG and oral mucosal immunopathology were strongly correlated in histopathological severity and T-cell population, suggesting a common initiation response explained by histopathological criteria of active disease driven by the T-cell infiltration in both tissues. However, the NIH cGVHD Development Consensus Project (2014) stated that non-synchronized disease stages should be considered when comparing oral mucosal and MSG immunohistopathology (13). With cGVHD progression in our cohort, CD8 T-cells and pathological scores showed negligible correlation, favoring different pathophysiologies (16, 17). This is interesting as salivary gland and oral mucosa cGVHD has been proposed as different pathophysiologies in clinical cohorts, but to our knowledge never been reported on an immunopathological level (16, 17, 26).

A diagnosis of oral cGVHD is not associated with hyposalivation or salivary gland dysfunction *per se*. Bassim et al. proposed a model of oral cGVHD as three separate diseases—mucosal lesions, salivary dysfunction, and peri-oral sclerosis—with only a 4% overlap between salivary gland dysfunction (UWS ≤0.2 mL/min) and other oral pathophysiologies, suggesting a morbid form of the disease (4, 17). This has been confirmed by other studies (26). Hypofunction of the salivary gland may present with mucosal GVHD-like pseudo manifestations, such as atrophic red, glossy sclerosis, or ulcerative lesions (9). In that context, it is interesting that a recent large-scale registry study found that almost 5% of patients with Sjögren's syndrome progressed to develop oral lichen planus, which are two different autoimmune disorders that oral cGVHD pathophysiology mimics (43). It could therefore be speculated that the oral mucous membrane could manifest secondary lichenoid-like disease following loss of mucosal integrity due to MSG pathology. In our cGVHD cohort, a significant positive correlation in immunolocalization of CD4 (*r* 0.71) and CD8 (*r* 0.69) was found between MSG and oral mucosal samples when clinically distinctive cGVHD lesions were analyzed. This may indicate synchronized inflammatory activity, where the pathway to oral mucosal immunological infiltrate is a result of persistent glandular dysfunction rather than true processes of mucosal GVHD immunopathology. This hypothesis is also strengthened by modest alignment in cGVHD histopathology (pathology score *r* 0.42 and grade *k* 0.33) within the same distinctive sample cohort, suggesting non-synchronized pathological processes. It should also be recognized that superficial mucoceles might be a consequence of mucosal cGVHD inflammation hindering saliva output, explaining a non-specific cGVHD manifestation of MSG in the state of oral mucosal cGVHD. Finally, CD4 staining demonstrated moderate-to-strong correlations across all clinical subpopulations, favoring a role for different subsets of T-helper cells and regulatory cells in distinct clinical settings.

Our retrospective cross-sectional study has several limitations, including potential bias in recruitment of study subjects and possible variations in the saliva circadian rhythm, which may have influenced the outcome. Furthermore, the influence on hyposalivation due to drug use and organ-specific GVHD manifestations was not considered due to incomplete registry data. Previous research has not found an association between medication use and number of medications and hyposalivation post-HCT (23, 26). Some study subgroups had a limited number of patients, which may explain the lack of significance, due to which some of the results should be taken with caution. This is particularly true for the UWS saliva groups, highlighting the need for rigorous, large-scale cohort studies that investigate UWS salivary levels. Immunosuppressive topical treatments might be a cofounding factor in correlation analysis, particularly in mild cGVHD cohorts, resulting in lower mucosal inflammation and alignment to MSG structures. However, the results from this large cohort reflect the same trends of hyposalivation reported by others and support the clinical theory that oral cGVHD represents different pathophysiologies (16, 17, 26).

We have demonstrated the lack of association between hyposalivation and GVHD but emphasized that severe cGVHD might present with stronger associations. Our data suggest that MAC-induced hyposalivation post-HCT recovers slower through the acute and chronic phases, with normalization to baseline levels not regained following the late chronic phase. Our comparable RIC data as well as UWS data require further investigation in modern transplant settings to fully explore the longitudinal effects post-HCT. Our findings not only add to the complex associations between MSG and oral mucosal tissue post-HCT but also explain how this may change under different circumstances. The immunopathological correlation observed during cGVHD onset, and lack of such correlation during cGVHD progression, validates previous clinical findings that salivary gland cGVHD and oral mucosal cGVHD represent two distinct conditions.

## Data Availability

The raw data supporting the conclusions of this article will be made available by the authors, without undue reservation.
