# Supplementary material for: Hyposalivation and salivary gland histopathology in graft-versus-host disease
Source: Front Oral Health. 2026 May 8;7:1812689. doi: 10.3389/froh.2026.1812689 (PMC13194362; doi:10.3389/froh.2026.1812689)
Supplement: Supplementary file 1 [file Supplementaryfile1.docx]

Supplementary Material

# Supplementary information (SI 1)

|  | **Time phase and salivary flow measurements** | | | | | |
| --- | --- | --- | --- | --- | --- | --- |
|  | **Acute time phase** | | **Early chronic time phase** | | **Late chronic time phase** | |
|  | **UWS** | **SWS** | **UWS** | **SWS** | **UWS** | **SWS** |
| **MAC** | | | | | | |
| Time | 65d (41-104d) | 77d (34-146d) | 225d (104-365d) | 197d (46-365d) | 625d (366-1854d) | 715d (366-1938d) |
| NO | 16 | 42 | 7 | 24 | 12 | 23 |
| Flow rate | 0.0300 | 0.2606 | 0.1271 | 0.8050 | 0.1825 | 0.6578 |
| SD | 0.0603 | 0.4129 | 0.1367 | 0.8782 | 0.1582 | 0.5108 |
| SE | 0.0151 | 0.0637 | 0.0517 | 0.1793 | 0.0457 | 0.1065 |
| vs prior-HCT | **p<0.0001** | **p<0.0001, ‡** | p=0.1539 | **p=0.0102, ‡** | p=0.2515 | **p=0.0002** |
| ***MAC - No acute/chronic GVHD*** | | | | | | |
| Time | 63d (42-91d) | 80d (35-137d) | 243d (120-365d) | 177d (46-365d) | 867d (380-1854d) | 666d (377-1854d) |
| NO | 4 | 11 | 2 | 8 | 4 | 7 |
| Flow rate | 0.0875 | 0.1341 | 0.1900 | 0.7450 | 0.1500 | 0.6314 |
| SD | 0.0957 | 0.1510 | 0.2404 | 0.8992 | 0.1291 | 0.6143 |
| SE | 0.0479 | 0.0455 | 0.1700 | 0.3179 | 0.0646 | 0.2322 |
| vs prior-HCT | p=0.6704 | **<0.0001** | p>0.9999 | p=0.1268 | p=0.8550 | **0.0207** |
| ***MAC –* *acute/chronic GVHD*** | | | | | | |
| Time | 66d (41-104d) | 76d (34-146d) | 219d (104-364d) | 210d (104-364d) | 731d (366-1938d) | 731d (366-1938d) |
| NO | 12 | 31 | 5 | 16 | 8 | 16 |
| Flow rate | 0.0108 | 0.3055 | 0.1020 | 0.8350 | 0.1988 | 0.6694 |
| SD | 0.0294 | 0.4663 | 0.1040 | 0.8958 | 0.1769 | 0.4809 |
| SE | 0.0085 | 0.0838 | 0.0465 | 0.2239 | 0.0626 | 0.1202 |
| vs prior-HCT | **p<0.0001** | **p<0.0001** | p=0.1696 | **p=0.0446** | p=0.6590 | **p=0.0015** |
| **RIC** | | | | | | |
| Time | 54d (42-70d) | 54d (42-70d) | 167d (136-183d) | 167d (136-183d) | 628d (378-926d) | 628d (378-926d) |
| NO | 3 | 4 | 3 | 3 | 4 | 4 |
| Flow rate | 0.3367 | 1.500 | 0.4333 | 1.800 | 0.2950 | 0.9750 |
| SD | 0.2811 | 0.6055 | 0.2517 | 0.9165 | 0.1047 | 0.4349 |
| SE | 0.1623 | 0.3028 | 0.1453 | 0.5292 | 0.0524 | 0.2175 |
| vs prior-HCT | NA | NA | NA | NA | NA | NA |

**Supplementary Data 1.** Descriptive and statistical data for salivary flow rate (ml/min) in respect to the time phases acute, early and late chronic, as well as the specified timepoint and range of the group data output. Data from patients with MAC-induced HCT are displayed as a composite group and separated into subgroups depending on presence of a/cGVHD or not. UWS - Unstimulated whole saliva, SWS - Stimulated whole saliva, MAC – Myeloablative conditioning, RIC - Reduced intensity conditioning, d – days, NO – Number of observations, SD - Std. Deviation, SE - Std. Error of Mean, ns – non-significant. Significance is shown with p-value in correspondence to Prior-HCT. Multiple comparison in MAC post-HCT was only found between SWS acute and early chronic phase: ‡ p=0.0468. Visualized data in RIC patients are not displayed with statistical comparisons as these groups were deemed with too small power and therefore statistical significance not reliable.

# Supplementary information (SI 2)


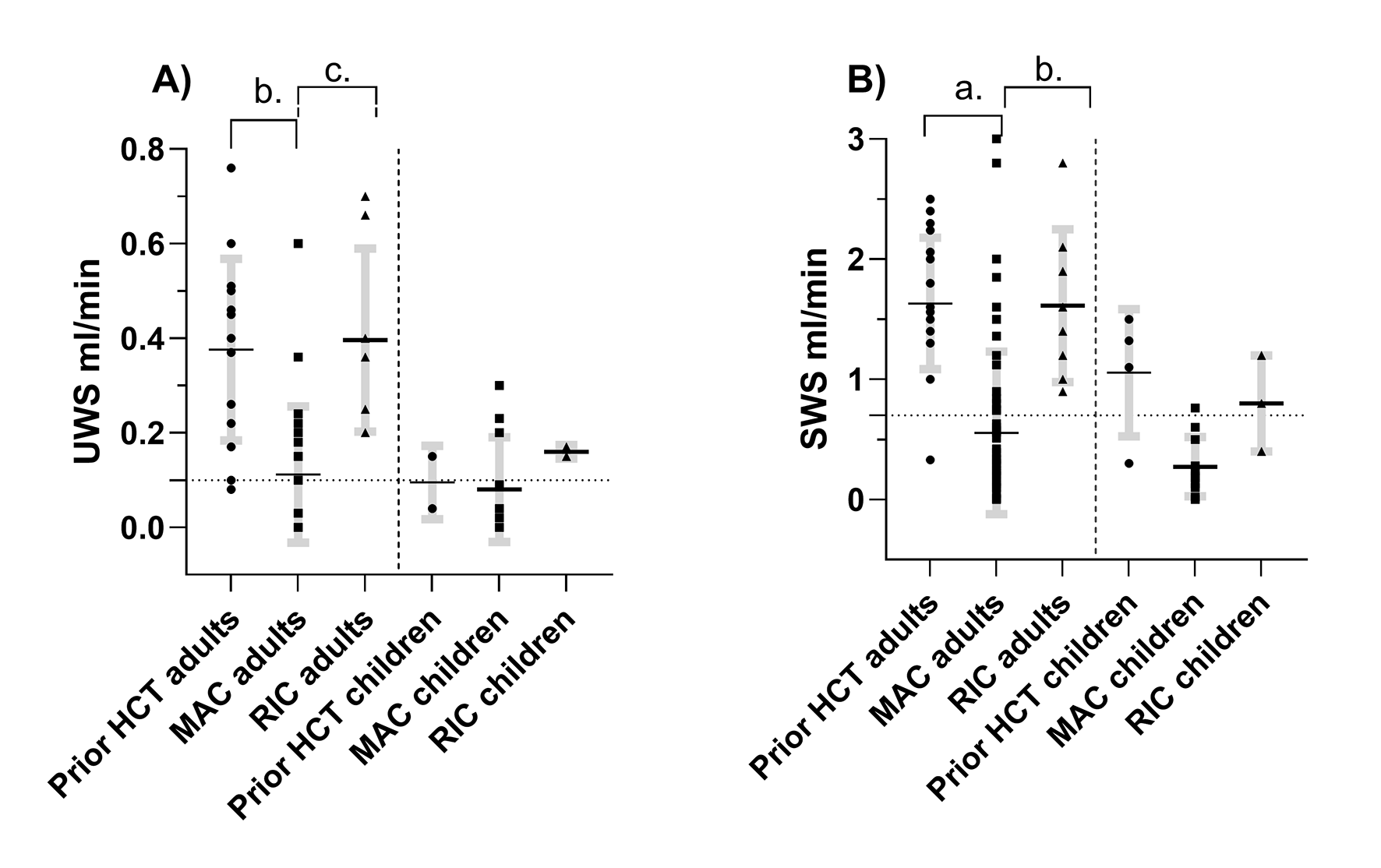


**Supplementary Figure 2.** Salivary flow rates are separated into adult and the child population. Diminishing levels of mean UWS and SWS were seen in all children compared to the adult cohort. Statistical differences were not found between child flowrates comparing Prior HCT, MAC och RIC. Horizontal dashed line indicates hyposalivation cut-off (UWS: ≤0.1 ml/min and SWS: ≤0.7 ml/min), whereas the dashed vertical line separates the adult and child cohorts. Statistical significance is indicated by a: p<0.0001, b: p<0.05 c: p<0.01, d: p <0.05. UWS - Unstimulated whole saliva, SWS - Stimulated whole saliva.

#
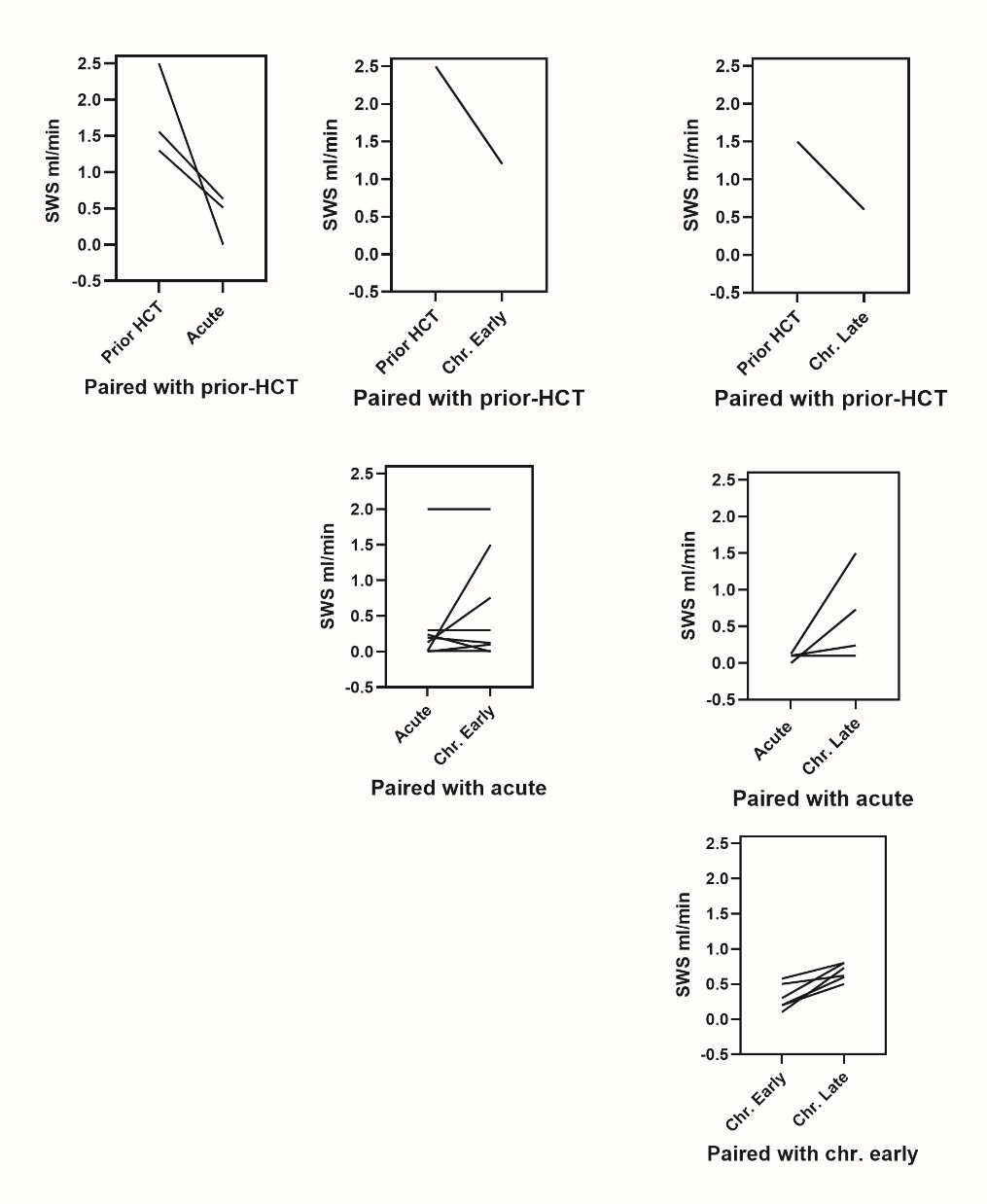
Supplementary information (SI 3)

**Supplementary Figure 3.** Spaghetti plots of repeated SWS measurements in 20 patients. Each line represents a patient with paired salivary flow rate between the phases of Prior-HCT, acute or early and late chronic time points. Two patients had three repeated measurements, but the majority only had one follow-up. In the top row, all flow rates paired with Prior-HCT are displayed, the middle row represents all flow rates paired with acute time phase, and subsequently paired measurements between early and late chronic are illustrated in the bottom row. UWS - Unstimulated whole saliva, SWS - Stimulated whole saliva.

# Supplementary information (SI 4)


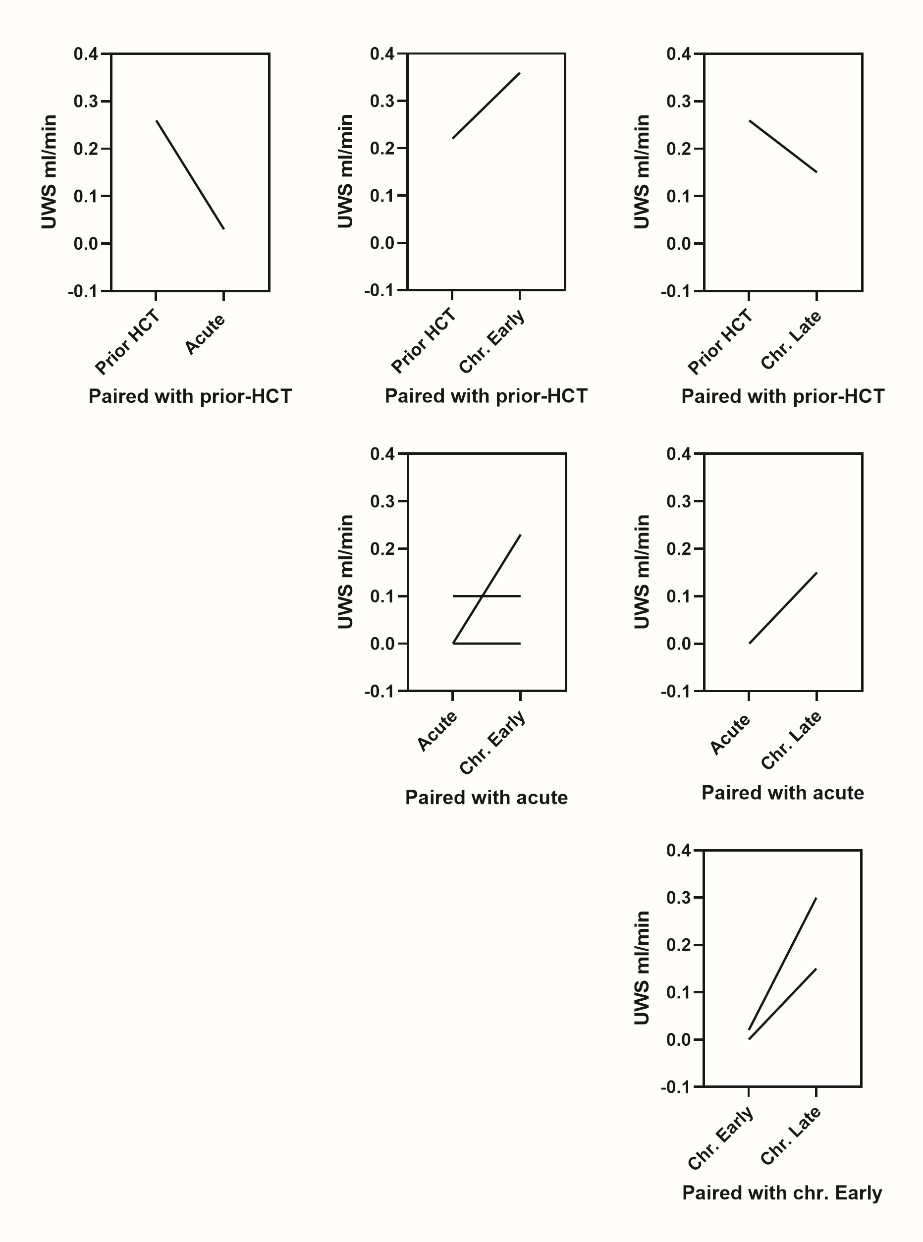


**Supplementary Data 4.** Spaghetti plots of repeated UWS measurements in seven patients. Each line represents a patient with paired salivary flow rate between the phases of Prior-HCT, acute or early and late chronic time. One patient had three repeated measurements, but the majority only had one follow-up. In the top row, all flow rates paired with Prior-HCT are displayed, the middle row represents all flow rates paired with acute time phase, and subsequently paired measurements between early and late chronic are illustrated in the bottom row. UWS - Unstimulated whole saliva, SWS - Stimulated whole saliva.

# Supplementary information (SI 5)

| **Distribution of intra-pathology agreement between MSG and oral mucosa** | | | | |
| --- | --- | --- | --- | --- |
| Post HCT-cohort |  | MSG G0-G1 | MSG GII | MSG GIII-IV |
|  | Mucosal G0-GI | 12 | 7 | 6 |
|  | Mucosal GII | 3 | 2 | 5 |
|  | Mucosal GIII-GIV | 4 | 4 | 14 |
| Post HCT-cohort MAC |  | MSG G0-G1 | MSG GII | MSG GIII-IV |
|  | Mucosal G0-GI | 8 | 6 | 6 |
|  | Mucosal GII | 3 | 2 | 4 |
|  | Mucosal GIII-GIV | 2 | 3 | 9 |
| Post HCT-cohort RIC |  | MSG G0-G1 | MSG GII | MSG GIII-IV |
|  | Mucosal G0-GI | 4 | 1 | 0 |
|  | Mucosal GII | 2 | 0 | 1 |
|  | Mucosal GIII-GIV | 2 | 1 | 5 |
| Oral mucosal cGVHD |  | MSG G0-G1 | MSG GII | MSG GIII-IV |
|  | Mucosal G0-GI | 7 | 4 | 3 |
|  | Mucosal GII | 2 | 2 | 5 |
|  | Mucosal GIII-GIV | 3 | 3 | 12 |
| Oral HCT controls |  | MSG G0-G1 | MSG GII | MSG GIII-IV |
|  | Mucosal G0-GI | 5 | 3 | 3 |
|  | Mucosal GII | 1 | 0 | 0 |
|  | Mucosal GIII-GIV | 1 | 1 | 2 |
| Diagnostic mucosal cGVHD |  | MSG G0-G1 | MSG GII | MSG GIII-IV |
|  | Mucosal G0-GI | 5 | 1 | 1 |
|  | Mucosal GII | 0 | 0 | 2 |
|  | Mucosal GIII-GIV | 3 | 2 | 8 |
| Distinctive mucosal cGVHD |  | MSG G0-G1 | MSG GII | MSG GIII-IV |
|  | Mucosal G0-GI | 3 | 3 | 2 |
|  | Mucosal GII | 1 | 2 | 3 |
|  | Mucosal GIII-GIV | 0 | 1 | 4 |
| Oral mucosal cGVHD 0-3 months |  | MSG G0-G1 | MSG GII | MSG GIII-IV |
|  | Mucosal G0-GI | 4 | 2 | 0 |
|  | Mucosal GII | 0 | 1 | 3 |
|  | Mucosal GIII-GIV | 0 | 2 | 8 |
| Oral mucosal cGVHD >3 months |  | MSG G0-G1 | MSG GII | MSG GIII-IV |
|  | Mucosal G0-GI | 3 | 2 | 3 |
|  | Mucosal GII | 2 | 1 | 3 |
|  | Mucosal GIII-GIV | 3 | 2 | 4 |

**Supplementary Data 5.** Intra-individual correlation was assessed between MSG and oral mucosa for post-HCT cohort (including MAC and RIC respectively) and oral HCT controls. Relationships were assessed between MSG and oral mucosa pathology scores using Spearman’s correlation to determine the r score, and weighted kappa (κ) for the NIH cGVHD grading (G0-GI, GII and GIII-GIV) (Presented in Table 2).
